# Systemic inflammation scores correlate with survival prognosis in patients with newly diagnosed brain metastases

**DOI:** 10.1038/s41416-020-01254-0

**Published:** 2021-01-21

**Authors:** Angelika M. Starzer, Ariane Steindl, Maximilian J. Mair, Carola Deischinger, Anika Simonovska, Georg Widhalm, Brigitte Gatterbauer, Karin Dieckmann, Gerwin Heller, Matthias Preusser, Anna S. Berghoff

**Affiliations:** 1 Department of Medicine I, Division of Oncology, Medical University of Vienna, Vienna, Austria; 2 Comprehensive Cancer Center, Medical University of Vienna, Vienna, Austria; 3 Department of Medicine III, Division of Endocrinology and Metabolism, Medical University of Vienna, Vienna, Austria; 4 Department of Neurosurgery, Medical University of Vienna, Vienna, Austria; 5 Department of Radiotherapy, Medical University of Vienna, Vienna, Austria

**Keywords:** CNS cancer, Prognostic markers, Outcomes research

## Abstract

**Background:**

Systemic inflammation measured by the neutrophil-to-lymphocyte ratio (NLR), leucocyte-to-lymphocyte ratio (LLR), platelet-to-lymphocyte ratio (PLR), monocyte-to-lymphocyte ratio (MLR) and CRP/albumin ratio (CRP/Alb) was shown to impact the survival prognosis in patients with extracranial solid cancer.

**Methods:**

One thousand two hundred and fifty patients with newly diagnosed brain metastases (BM) were identified from the Vienna Brain Metastasis Registry.

**Results:**

PLR and CRP/Alb were higher in patients with progressive extracranial disease and lower in patients with no evidence of extracranial disease. Lower NLR (cut-off = 5.07; 9.3 vs. 5.0 months), LLR (cut-off = 5.76; 10.0 vs. 5.3 months), PLR (cut-off = 335; 8.0 vs. 3.8 months), MLR (cut-off = 0.53; 6.0 vs. 3.5 months) and CRP/Alb (cut-off = 2.93; 8.5 vs. 3.7 months; *p*_adj_ < 0.05) were associated with longer overall survival (OS). In multivariate analysis with graded prognostic assessment (hazard ratio (HR) 1.45; 95% confidence interval (CI): 1.32–1.59; *p*_adj_ = 1.62e − 13_)_, NLR (HR 1.55; 95% CI: 1.38–1.75; *p*_adj_ = 1.92e − 11), LLR (HR 1.57; 95% CI: 1.39–1.77; *p*_adj_ = 1.96e − 11_)_, PLR (HR 1.60; 95% CI: 1.39–1.85; *p*_adj_ = 2.87955e − 9), MLR (HR 1.41; 95% CI: 1.14–1.75; *p*_adj_ = 0.027) and CRP/Alb (HR 1.83; 95% CI: 1.54–2.18; *p*_adj_ = 2.73e − 10) remained independent factors associated with OS at BM diagnosis.

**Conclusions:**

Systemic inflammation, measured by NLR, LLR, PLR, MLR and CRP/Alb, was associated with OS in patients with BM. Further exploration of immune modulating therapies is warranted in the setting of BM.

## Background

Brain metastases (BMs) are a frequent and life limiting complication in solid cancers.^1^ Immune checkpoint inhibitor-based therapies have shown activity in patients with BM.^1–4^ However, the clinical efficacy is higher in patients with asymptomatic BM compared to patients suffering from neurological symptoms in need for steroid treatment.^3^ Indeed, immune responses are tightly controlled in the brain in order to avoid any harmful damage to this sensitive organ.^5^ Previously, the density of intratumoural T cells was described as a favourable prognostic factor in BM patients underscoring the prognostic importance of cancer–immune system interactions also in patients with BM.^6^ Recent insights into the cancer–immune system interactions further stressed the importance of systemic inflammation in addition to the local inflammatory characteristics.^7^ Systemic inflammatory scores, including neutrophil-to-lymphocyte ratio (NLR), leucocyte-to-lymphocyte ratio (LLR), platelet-to-lymphocyte ratio (PLR), monocyte-to-lymphocyte ratio (MLR) and C-reactive protein/albumin ratio (CRP/Alb), were shown to impact tumour control and in consequence survival prognosis of patients with extracranial metastatic cancer.^8–12^ Moreover, markers of systemic inflammation, including pre-treatment CRP, NLR and MLR, were recently shown to be associated with the progression-free survival (PFS) and overall survival (OS) in patients treated with immune checkpoint inhibitor-based therapy.^13–15^ However, the prognostic impact of systemic inflammation was not yet investigated in a comprehensive real-life cohort of BM patients. Therefore, we aimed to analyse systemic inflammation measured by the NLR, LLR, PLR, MLR and CRP/Alb and their correlation with survival prognosis in a large real-life cohort of patients with BM.

## Methods

### Patients and data collection

Patients with newly diagnosed BM from solid tumours and treated at the Medical University of Vienna between 1990 and 2019 were identified from the *Vienna Brain Metastasis Registry*. Investigated markers of systemic inflammation included the NLR, LLR, PLR, MLR and CRP/Alb at the time of BM diagnosis ±14 days. Steroid therapy at BM diagnosis was defined as ±14 days from diagnosis of BM. Data closest to the diagnosis of BM was chosen for analysis. Synchronous diagnosis of BM with primary cancer or progressive extracranial disease (new or growing extracranial lesions) is defined as ±30 days from BM diagnosis. The graded prognostic assessment (GPA) score of our patient cohort was calculated based on clinical parameters, which include age, Karnofsky performance score, number of BM and the status of the extracranial disease, as previously described.^16^ The ACCI was calculated as previously published.^17^ For each included comorbidity a score was given. A total score was calculated by the sum of scores for each comorbidity and for each decade starting with 50 years 1 point was added (e.g. addition of 1 point for age group 50–59 years).^11^ The studied patient cohort was treated independently by multidisciplinary teams according to good clinical practice guidelines. This project was approved by the ethics committee of the Medical University of Vienna (078/2004).

### Statistical analysis

Cancer entities occurring <10 times in the entire cohort were summarised under the primary cancer entity “others”. The Kolmogornov–Smirnov test was used to test for data normality. Differences between groups were assessed using the Kruskal–Wallis test. Correlations of metric variables were determinated using the Spearman’s rho, while a correlation coefficient of *ρ* > 0.7 was interpreted as strong correlation, 0.7 ≥ *ρ* > 0.5 as medium correlation, 0.5 ≥ *ρ* > 0.3 as weak correlation and <0.3 as no correlation. OS from diagnosis of BM was defined as time from radiological diagnosis of BM until death or last follow-up. Patient stratification cut-offs for survival analyses were calculated according to the maximally selected rank statistics using the R package *maxstat* that iteratively tests all possible cut-points to identify the value with the maximum rank statistics for optimal group stratification for survival analyses.^18,19^ The Kaplan–Meier product limit method was used to illustrate survival times and log-rank tests were calculated to estimate survival differences between groups. Survival analyses were calculated using the R packages *survival* and *survminer*.^20,21^ A multivariate analysis using the Cox proportional hazard model was applied to adjust for the GPA score as an established prognostic assessment.^16^ *P* values were Bonferroni adjusted for 27 applied statistical tests resulting in adjusted *p* values (*p*_adj_) for each statistical test. A two-tailed *p*_adj_ < 0.05 was considered to indicate statistical significance. Statistical analyses were performed using the Statistical Package for the Social Sciences (SPSS®) 23.0 software (SPSS Inc., Chicago, IL, USA) and R (R Foundation for Statistical Computing, Vienna, Austria).

## Results

### Patients characteristics

One thousand two hundred and fifty patients (662/1250, 53% males; 588/1250, 47% females) with a median age of 62 years (range 23–91) at diagnosis of BM were included in the analysis. Five hundred and seventy-one of 1250 (45.7%) patients were diagnosed with BM simultaneously with diagnosis of the primary tumour. One hundred and eight of 1250 (8.6%) patients showed no evidence of extracranial disease at BM diagnosis, while stable extracranial disease at BM diagnosis was evident in 233/1250 (18.6%) patients. Three hundred and thirty-eight of 1250 (27.0%) patients presented with synchronous progressive extracranial disease. One thousand one hundred and sixty-nine of 1250 (93.5%) patients were locally treated with neurosurgical resection (7%), radiotherapy (77.8%) or a combination of surgery and radiotherapy (8.7%) of BM, while 34/1250 (2.7%) of patients received systemic therapy or best supportive care only (47/1250, 3.8%). Patients receiving systemic therapy as first-line treatment for BM showed the longest OS from diagnosis of BM (15 months), followed by a combinational therapy of surgery plus radiotherapy (14 months), surgery only (7 months), radiotherapy only (6 months) and best supportive care (1 month; log-rank test; *p*_adj_ = 1.6e − 20). Median survival in the entire cohort was 6 months (range 0–178 months; Table 1). GPA class showed a significant association with survival prognosis from diagnosis of BM in univariate analysis (hazard ratio (HR) 1.47; 95% confidence interval (CI): 1.35–1.62; *p*_adj_ = 2.10e − 15; Cox regression model); Supplementary Fig. 1).

**Table 1:** Patients characteristics.

Characteristics	*n* = 1250	100%
Age at diagnosis of BM, years		
Median (range)	62 (23–91)	
Sex		
Male	662	53
Female	588	47
Cancer entity		
Lung cancer	994	79.5
Breast cancer	86	6.9
Melanoma	106	8.5
Renal cell carcinoma	7	0.6
Colorectal cancer	11	0.9
CUP	10	0.8
Others	36	2.9
Surgery of the primary tumour		
Yes	359	28.7
No	891	71.3
Radiotherapy to the primary tumour site		
Yes	221	17.7
No	1029	82.3
Adjuvant chemotherapy		
Yes	346	27.7
No	904	72.3
KPS		
Median (range)	80 (0–100)	
GPA class		
Class I	41	3.3
Class II	121	9.7
Class III	761	60.9
Class IV	327	26.2
Status of extracranial disease		
Synchronous diagnosis of BM at cancer diagnosis	571	45.7
No evidence of extracranial disease	108	8.6
Stable disease	233	18.6
Progressive disease	338	27.0
Chemotherapy before diagnosis of BM		
Yes	581	46.5
No	669	53.5
Steroid treatment at BM diagnosis		
Yes	479	38.3
No	728	58.2
NA	43	3.4
First-line treatment of BM		
Surgery	87	7.0
Radiotherapy total	973	77.8
(GK/WBRT/GK + WBRT)	(597/280/96)	(47.8/22.4/7.6)
(Radiotherapy within 14 days of BM diagnosis, *n* = 973*)	(432)	(44.4*)
Combinational local therapy (surgery + radiotherapy)	109	8.7
Systemic therapy	34	2.7
BSC	47	3.8
Overall survival, months		
Median (range)	6 (0–178)	
Alive	151	12.1
Deceased	1099	87.9

*BM* brain metastasis, *CUP* cancer of unknown primary, *KPS* Karnofsky performance status, *GPA* graded prognostic assessment, *NA* not available, *GK* gamma knife, *WBRT* whole-brain radiotherapy, *BSC* best supportive care.

*Total number of patients having had radiotherapy.

### Systemic inflammation in patients with newly diagnosed brain metastases

NLR, LLR and PLR were available in all included patients, while MLR was available in 379/1250 (30.3%) and CRP/Alb in 601/1250 (48.1%) patients (Table 2). No differences in NLR, LLR, PLR, MLR or CRP/Alb according to primary tumour type was observed (*p*_adj_ > 0.05; Kruskal–Wallis test; Supplementary Fig. 2).

**Table 2:** Systemic inflammation scores.

Characteristics	Median	Range
Leucocytes G/L, *n* = 1250	8.69	0.89–48.8
Neutrophils G/L, *n* = 1250	6.15	0.24–66.0
Lymphocytes G/L, *n* = 1250	1.3	0.1–26.5
Platelets G/L, *n* = 1250	272.0	2.0–894.0
Monocytes G/L, *n* = 379	0.7	0.01–2.42
CRP, *n* = 1190	1.22	0–43.7
Albumin, *n* = 612	39.8	15.7–53.38
NLR, *n* = 1250	4.76	0.07–98.0
LLR, *n* = 1250	6.39	0.12–997.5
PLR, *n* = 1250	207.57	3.08–1675.0
MLR, *n* = 379	0.52	0.01–3.06
CRP/Alb, *n* = 601	2.41	0.02–121.64

*CRP* C-reactive protein, *NLR* neutrophil-to-lymphocyte ratio, *LLR* leucocyte-to-lymphocyte ratio, *PLR* platelet-to-lymphocyte ratio, *MLR* monocyte-to-lymphocyte ratio, *CRP/Alb* C-reactive protein/albumin ratio.

There were no correlations of systemic inflammation scores with age nor with KPS nor with age-adjusted Charlson-comorbidity index at BM diagnosis (Spearman correlation coefficient <0.3).

Twenty-one of 1250 (1.7%) patients in this cohort showed a history of autoimmune disease. There was no correlation of investigated inflammation markers at BM diagnosis and history of autoimmune disease (Mann–Whitney *U* test, *p*_adj_ > 0.05).

**Fig. 1:**
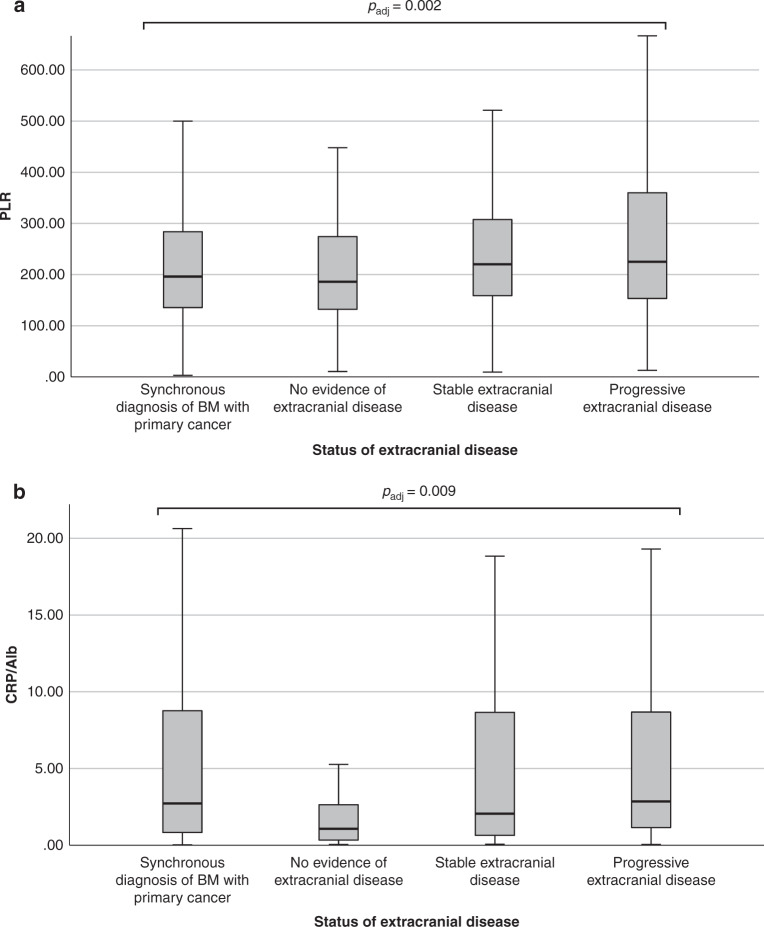
Levels of systemic inflammation scores according to the status of the extracranial disease. **a** Median platelet-to-lymphocyte ratio (PLR) and **b** median C-reactive protein/albumin ratio (CRP/Alb) according to the status of the extracranial disease are highest in patients with progressive disease (*p* < 0.001; Kruskal–Wallis test). Bar graphs + confidence intervals (CIs) are shown.

PLR and CRP/Alb showed significant differences according to the status of the extracranial disease. PLR was highest in patients with progressive extracranial disease (median PLR = 225.0), followed by patients with stable disease (median PLR = 220.0) and patients with synchronous diagnosis of BM with the primary cancer (median PLR = 196.1) and lowest in patients with no evidence of extracranial disease at BM diagnosis (median = PLR 189.1; *p*_adj_ = 0.002; Kruskal–Wallis test; Fig. 1a). CRP/Alb was highest in patients with progressive extracranial disease (median CRP/Alb = 2.85), followed by patients with synchronous diagnosis of BM with the primary cancer (median CRP/Alb = 2.73), followed by patients with stable disease (median CRP/Alb = 2.06) and lowest in patients with no evidence of extracranial disease (median CRP/Alb = 1.07; *p*_adj_ = 0.009; Kruskal–Wallis test; Fig. 1b). NLR, LLR and MLR did not significantly differ depending on the status of the extracranial disease (*p*_adj_ > 0.05; Kruskal–Wallis test).

**Fig. 2:**
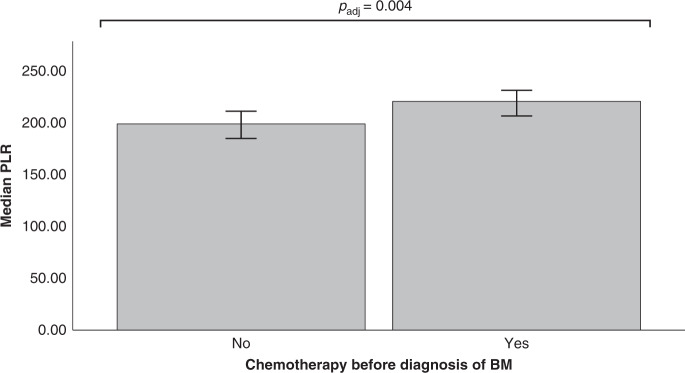
Levels of platelet-to-lymphocyte ratio according to prior chemotherapy before diagnosis of BM. Median platelet-to-lymphocyte ratio (PLR) according to prior application of chemotherapy before diagnosis of BM (median PLR 220.0) compared to patients without prior chemotherapy (median PLR 198.33; *p* = 0.004; Mann–Whitney *U* test). Bar graphs + CIs are shown.

Patients with prior application of chemotherapy presented with a higher median PLR (median PLR 220.0) compared to chemotherapy-naive patients (median PLR 198.52; *p*_adj_ = 0.004; Mann–Whitney *U* test; Fig. 2). No differences in patients with or without prior chemotherapy were observed concerning the NLR, LLR, MLR and CRP/Alb (*p* > 0.05; Mann–Whitney *U* test).

Four hundred and seventy-nine of 1250 (38.3%) BM patients received steroid treatment at BM diagnosis. No significant differences in investigated inflammation scores were observed in patients treated with steroids compared to patients without steroid therapy (*p*_adj_ > 0.05; Mann–Whitney *U* test).

Twenty-two of 1250 patients (1.8%) in this cohort were treated with immune checkpoint inhibitors before the diagnosis of BM. No significant difference of inflammation markers at BM diagnosis according to therapy with immune checkpoint inhibitors prior to diagnosis of BM was observed (*p*_adj_ > 0.05; Mann–Whitney *U* test).

### Correlation of systemic inflammation markers with survival prognosis

**Fig. 3:**
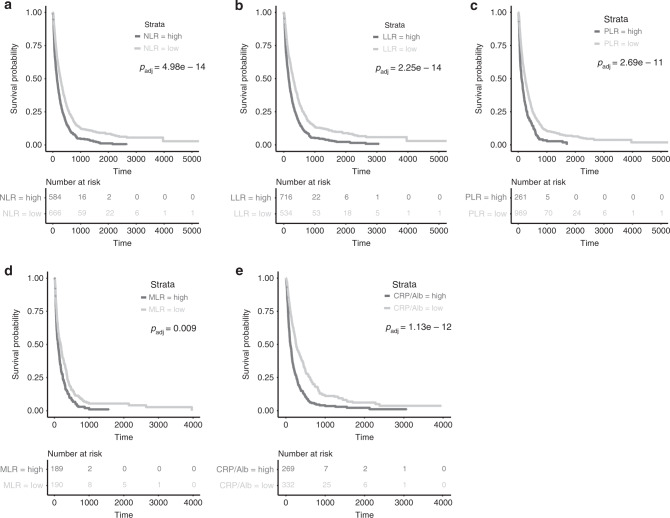
Overall survival from diagnosis of BM according to markers of systemic inflammation. **a** Neutrophil-to-lymphocyte ratio (NLR) (*p*_adj_ = 4.98e − 14; log-rank test), **b** Leucocyte-to-lymphocyte ratio (LLR) (*p*_adj_ = 2.25e − 14; log-rank test), **c** Platelet-to-lymphocyte ratio (PLR) (*p*_adj_ = 2.69e − 11; log-rank test), **d** Monocyte-to-lymphocyte ratio (MLR) (*p*_adj_ = 0.009; log-rank test), **e** C-reactive protein/albumin ratio (CRP/Alb) (*p*_adj_ = 1.13e − 12; log-rank test).

Lower NLR (cut-off = 5.07) was associated with a significantly longer OS with 9.3 months compared to 5.0 months in patients with a higher NLR (*p*_adj_ = 4.98e − 14; log-rank test; Fig. 3a). Further, patients with lower LLR (cut-off = 5.76; 10.0 vs. 5.3 months, *p*_adj_ = 2.25e − 14; log-rank test; Fig. 3b), lower PLR (cut-off = 335; 8.0 vs. 3.8 months; *p*_adj_ = 2.69e − 11; log-rank test; Fig. 3c), lower MLR (cut-off = 0.53; 6.0 vs. 3.5 months; *p*_adj_ = 0.009; log-rank test; Fig. 3d) and lower CRP/Alb (cut-off = 2.93; 8.5 vs. 3.7 months; *p*_adj_ = 1.13e − 12; log-rank test; Fig. 3e) presented with a more favourable survival prognosis.

In a multivariate model adjusting for GPA (in 1250/1250 patients), NLR (HR 1.55; 95% CI: 1.38–1.75; *p*_adj_ = 1.92e − 11; Cox regression model), LLR (HR 1.57; 95% CI: 1.1.39–1.1.77; *p*_adj_ = 1.96e − 11; Cox regression model), PLR (HR 1.60; 95% CI: 1.39–1.85; *p*_adj_ = 2.88e − 9; Cox regression model), MLR (HR 1.41; 95% CI: 1.14–1.75; *p*_adj_ = 0.027; Cox regression model) and CRP/Alb (HR 1.83; 95% CI: 1.54–2.18; *p*_adj_ = 2.73e − 10; Cox regression model) remained independent factors associated with OS after diagnosis of BM (Table 3). In adjusting for the DS-GPA (in 1239/1250 patients with the DS-GPA available), the NLR (HR 1.45; 95% CI: 1.28–1.64; *p*_adj_ = 1.30e − 7; Cox regression model), LLR (HR 1.43; 95% CI: 1.26–1.63; *p*_adj_ = 5.46e − 7; Cox regression model), PLR (HR 1.54; 95% CI: 1.33–1.79; *p*_adj_ = 2.32e − 7; Cox regression model), MLR (HR 1.40; 95% CI: 1.13–1.74; *p*_adj_ = 0.05; Cox regression model) and CRP/Alb (HR 1.71; 95% CI: 1.42–2.05; *p*_adj_ = 2.45e − 7; Cox regression model) remained independent factors associated with OS after diagnosis of BM.

**Table 3:** Multivariate analysis.

	Overall survival		Cox regression
	HR	95% CI	*p*_adj_
GPA class	1.46	1.32–1.59	1.62e − 13
NLR	1.55	1.38–1.75	1.92e − 11
LLR	1.57	1.39–1.77	1.96e − 11
PLR	1.60	1.39–1.85	2.88e − 9
MLR	1.41	1.14–1.75	0.027
CRP/Alb	1.83	1.54–2.18	2.73e − 10

*HR* hazard ratio, *CI* confidence interval, *GPA* graded prognostic assessment, *NLR* neutrophil-to-lymphocyte ratio, *LLR* leucocyte-to-lymphocyte ratio, *PLR* platelet-to-lymphocyte ratio, *MLR* monocyte-to-lymphocyte ratio, *CRP/Alb* C-reactive protein/albumin ratio.

## Discussion

Systemic inflammation scores correlated with survival prognosis in our cohort of advanced cancer patients with BM. The routinely and easily accessible NLR, LLR, PLR, MLR and CRP/Alb had independent prognostic impact in addition to the established GPA, suggesting that also in the advanced event of BM, flourishing systemic inflammatory processes are negatively associated with the course of cancer disease.

PLR and CRP/Alb were significantly higher in patients with simultaneous progressive extracranial disease at BM diagnosis compared to patients with a stable extracranial disease and patients with synchronous diagnosis of BM. In contrast, NLR, LLR and MLR did not correlate with the status of the extracranial disease. In consequence, PLR and CRP/Alb might be more determined by the status of the systemic disease than NLR, LLR and MLR. The acute-phase protein CRP increases during systemic inflammatory processes while the albumin production is reduced as an amino acid sparing mechanism.^22^ The Glasgow Prognostic Score includes the CRP/Alb as a prognostic marker for survival independent of cancer entity or disease stage.^23^ Previously, activated platelets were shown to stimulate inflammatory processes by the release of vascular endothelial growth factor and platelet-derived growth factor, which mediate the extravasation and migration of leucocytes.^24^ Further, platelets are postulated to contribute to cancer dissemination by depleting natural killer cells and impairing their cytotoxic activity.^25^ The cell-based scores NLR, LLR and MLR might reflect a more immediate impact of inflammation as neutrophils are the first effector immune cells recruited in case of acute inflammation followed by monocytes.^26^ Indeed, normalisation of NLR after one cycle of chemotherapy was described to result in improved PFS in colorectal cancer and mesothelioma patients.^27,28^ Previous chemotherapeutic treatments potentially impact the investigated inflammatory signatures. Indeed, PLR was higher in patients with previous treatment, but NLR, LLR, MLR and CRP/Alb were not impacted by previous treatments. Therefore, the investigated systemic inflammatory scores give a prognostic relevant insight into the systemic inflammatory status also in pretreated BM patients.

The investigated systemic inflammation scores NLR, LLR, PLR, MLR and CRP/Alb presented with sustained prognostic impact independent from the GPA. The variable set included in the GPA does only include clinical variables like age, number of BM, Karnofsky performance score and status of the extracranial disease.^16^ Previously, we reported that the addition of laboratory parameters, included in the LabBM score, provide a more precise prognostic prediction than the GPA alone.^29^ A precise survival prediction is of particular importance in BM patients as treatment decisions have to be taken in the careful balance between efficacy and short/long-term side effects in a palliative setting.^30^ The CRP value was the only inflammatory marker included in the LabBM score. However, systemic inflammation is of growing importance in extracranial malignancies as, besides the prognostic impact, pre-treatment NLR, MLR, PLR and CRP were recently shown to be associated with PFS and OS in melanoma, non-small cell lung cancer, renal cell carcinoma, breast and head and neck cancer patients treated with immune checkpoint inhibitor therapy.^13–15,31,32^ Immune checkpoint inhibitors have increasing clinical importance in BM patients as first clinical trials strongly support the application in selected patient populations with asymptomatic newly diagnosed BM.^33,34^ None of the patients in the present series were treated with immune checkpoint inhibitors after diagnosis of BM and, therefore, future trials should investigate whether systemic inflammatory scores could have predictive potential for the response to immune checkpoint inhibitors in the BM population.

Although we investigated a particularly large, real-life cohort, the retrospective study argues for careful interpretation of the obtained data. The comprehensive set of clinical data allowed us to statistically investigate the impact of the primary tumour type, the status of the extracranial disease as well as the previous treatments on the investigated systemic inflammatory scores. The obtained data further reflects everyday practice as values ±14 days from diagnosis of BM were included and certain fluctuation in the blood values could occur in this time frame. Nevertheless, the independent association in addition to GPA supports the prognostic impact. Prospective studies would be warranted to validate the observed prognostic impact of systemic inflammation in newly diagnosed BM patients.

## Conclusions

In conclusion, we show that systemic inflammation scores correlate with survival prognosis in a large real-life cohort of patients with advanced cancer and brain metastases. Further, our data suggest that activated systemic inflammation possibly impacts cancer progression also in the setting of BM. Future trials investigating immune modulating therapies should therefore also consider monitoring systemic inflammation scores and their predictive value to outcome to immunotherapy, eventually finding new predictive markers for a personalised immunotherapy approach in BM patients.

## Supplementary information

Supplementary information

## Data Availability

The data supporting the results in this manuscript is saved at a server of the Medical University of Vienna.
