# Supplementary material for: Systemic inflammation scores correlate with survival prognosis in patients with newly diagnosed brain metastases
Source: Br J Cancer. 2021 Jan 21;124(7):1294–300. doi: 10.1038/s41416-020-01254-0 (PMC8007827; doi:10.1038/s41416-020-01254-0)
Supplement: Supplementary file 1 — Supplementary information [file 41416_2020_1254_MOESM1_ESM.docx]

**Supplementary information to “Systemic inflammation scores correlate with survival prognosis in patients with newly diagnosed brain metastases“ by Starzer et al.**

**Supplementary Figure 1:** Overall survival from diagnosis of BM according to GPA Class 1 to 4 (p_adj_= 3.32e-16; log rank test).

**Supplementary Figure 2:** **A** Median neutrophil-to-lymphocyte ratio (NLR), **B** median leucocyte-to-lymphocyte ratio (LLR), **C** median platelet-to-lymphocyte ratio (PLR), **D** median monocyte-to-lymphocyte ratio (MLR), **E** median C-reactive protein/Albumin ratio (CRP/Alb) according to primary tumor entities (p>0.05; Kruskal-Wallis test). Bar graphs + CI are shown, CUP cancer of unknown primary.
